# Genetic determinants of FOXM1 overexpression in epithelial ovarian cancer and functional contribution to cell cycle progression

**DOI:** 10.18632/oncotarget.4546

**Published:** 2015-07-16

**Authors:** Carter J. Barger, Wa Zhang, Joanna Hillman, Aimee B. Stablewski, Michael J. Higgins, Barbara C. Vanderhyden, Kunle Odunsi, Adam R. Karpf

**Affiliations:** ^1^ Eppley Institute and Fred & Pamela Buffett Cancer Center, University of Nebraska Medical Center, Omaha, NE, 68198, USA; ^2^ Department of Molecular and Cellular Biology, Roswell Park Cancer Institute, Buffalo, NY, 14263, USA; ^3^ Department of Cellular and Molecular Medicine, University of Ottawa, Ottawa, Ontario, K1H 8M5, Canada; ^4^ Department of Immunology, Roswell Park Cancer Institute, Buffalo, NY, 14263, USA; ^5^ Department of Gynecologic Oncology, Roswell Park Cancer Institute, Buffalo, NY, 14263, USA; ^6^ Center for Immunotherapy, Roswell Park Cancer Institute, Buffalo, NY, 14263, USA

**Keywords:** FOXM1, epithelial ovarian cancer, p53, Rb, E2F1

## Abstract

The FOXM1 transcription factor network is frequently activated in high-grade serous ovarian cancer (HGSOC), the most common and lethal subtype of epithelial ovarian cancer (EOC). We used primary human EOC tissues, HGSOC cell lines, mouse and human ovarian surface epithelial (OSE) cells, and a murine transgenic ovarian cancer model to investigate genetic determinants of FOXM1 overexpression in EOC, and to begin to define its functional contribution to disease pathology. The Cancer Genome Atlas (TCGA) data indicated that the *FOXM1* locus is amplified in ~12% of HGSOC, greater than any other tumor type examined, and that *FOXM1* amplification correlates with increased expression and poor survival. In an independent set of primary EOC tissues, *FOXM1* expression correlated with advanced stage and grade. Of the three known *FOXM1* isoforms, *FOXM1c* showed highest expression in EOC. In murine OSE cells, combined knockout of *Rb1* and *Trp53* synergistically induced FOXM1. Consistently, human OSE cells immortalized with SV40 Large T antigen (IOSE-SV) had significantly higher FOXM1 expression than OSE immortalized with hTERT (IOSE-T). FOXM1 was overexpressed in murine ovarian tumors driven by combined *Rb1*/*Trp53* disruption. FOXM1 induction in IOSE-SV cells was partially dependent on E2F1, and FOXM1 expression correlated with E2F1 expression in human EOC tissues. Finally, FOXM1 functionally contributed to cell cycle progression and relevant target gene expression in human OSE and HGSOC cell models. In summary, gene amplification, p53 and Rb disruption, and E2F1 activation drive FOXM1 expression in EOC, and FOXM1 promotes cell cycle progression in EOC cell models.

## INTRODUCTION

Approximately 70% of EOC cases are diagnosed at advanced stage; long-term survival for these patients is poor and has not improved significantly in the past three decades [1, 2]. Current clinical management of EOC is surgical debulking and adjuvant chemotherapy using a platinum-taxane doublet. While the majority EOC patients are initially responsive to chemotherapy, most patients relapse and current second line therapies are not curative. Increased knowledge of the pathological and genetic underpinnings of EOC and HGSOC, its most common and lethal subtype, are likely to lead to advances in diagnosis and treatment [3]. For example, TCGA recently reported mRNA and miRNA expression, DNA copy number alterations (CNA), DNA promoter methylation, and mutational data for HGSOC, which led to classification into sub-groups based on these molecular criteria [4]. CNA is prominent in HGSOC, and occurs at a higher frequency than in any other TCGA-profiled tumor type [4–6]. It was also notable that *TP53* was mutated in virtually all HGSOC, suggesting p53 as a “gatekeeper” for this disease [4]. Other tumor suppressors and oncogenes implicated in HGSOC include BRCA1/2, Rb, PI3K, Ras, and CCNE1 [4, 7–9]. Finally, FOXM1 pathway activation is a highly frequent alteration in HGSOC, second only to *TP53* mutation [4].

FOXM1 is a member of the Forkhead box (FOX) transcription factor family, which is unified by a conserved winged helix DNA binding motif [10]. The binding specificity of FOXM1 relative to other family members is in part achieved via an atypical chromatin interaction mechanism in which FOXM1 is bridged to DNA by the Myb-MuvB (MMB) transcriptional activator complex [11]. At least two important biological pathways are influenced by the transcriptional activity of FOXM1: cell cycle (G1-S and G2-M transitions), and DNA damage (homologous recombination DNA repair) [10, 12]. FOXM1 is overexpressed and activated in many human cancers and possesses oncogenic activity *in vitro* and *in vivo* [12]. Mechanisms accounting for FOXM1 overexpression in cancer cells and tissues are diverse and include p53, Rb, and FOXO3 loss [13–16], Myc, HIF-1, Gli1, SP1, STAT3 and E2F activation [17–22], and gene amplification [23].

Human *FOXM1* has 10 exons with alternative splicing of exons Va (A1) and VIIa (A2), giving rise to three FOXM1 variants: FOXM1a, FOXM1c, and FOXM1b. FOXM1a contains exons Va and VIIa, with the latter disrupting the transactivation domain, making this isoform transcriptionally inactive [24]. FOXM1 expression was reported to be restricted to dividing cells with onset of expression at late G1 and peak expression at G2-M [25, 26]. FOXM1 protein is additionally regulated throughout the cell cycle via phosphorylation [10]. Once activated, FOXM1 can promote cell cycle progression through transactivation of target genes, leading to progression through both G1-S [27–29] and G2-M [30–32] checkpoints.

The goal of the current study was to begin to define the genetic determinants of FOXM1 overexpression in EOC, to analyze its expression during disease progression, and to investigate its role in EOC cell cycle progression. For this task, we utilized publically available EOC databases, primary human EOC tissues, immortalized ovarian surface epithelial (OSE) cell models (murine and human), a transgenic murine ovarian cancer model, and human HGSOC cell lines. Together, our data implicate gene amplification, Rb and p53 inactivation, and E2F1 activation in FOXM1 overexpression in EOC. Among *FOXM1* isoforms, *FOXM1c* showed highest expression in EOC cells and tumors. FOXM1 was overexpressed in late-stage, high-grade disease, and *FOXM1* gene amplification correlated with reduced HGSOC survival. Finally, we demonstrated that FOXM1 contributes to cell cycle progression in OSE and HGSOC cell models.

## RESULTS

### *FOXM1* gene amplification correlates with increased *FOXM1* expression and reduced survival in HGSOC

**Figure 1:**
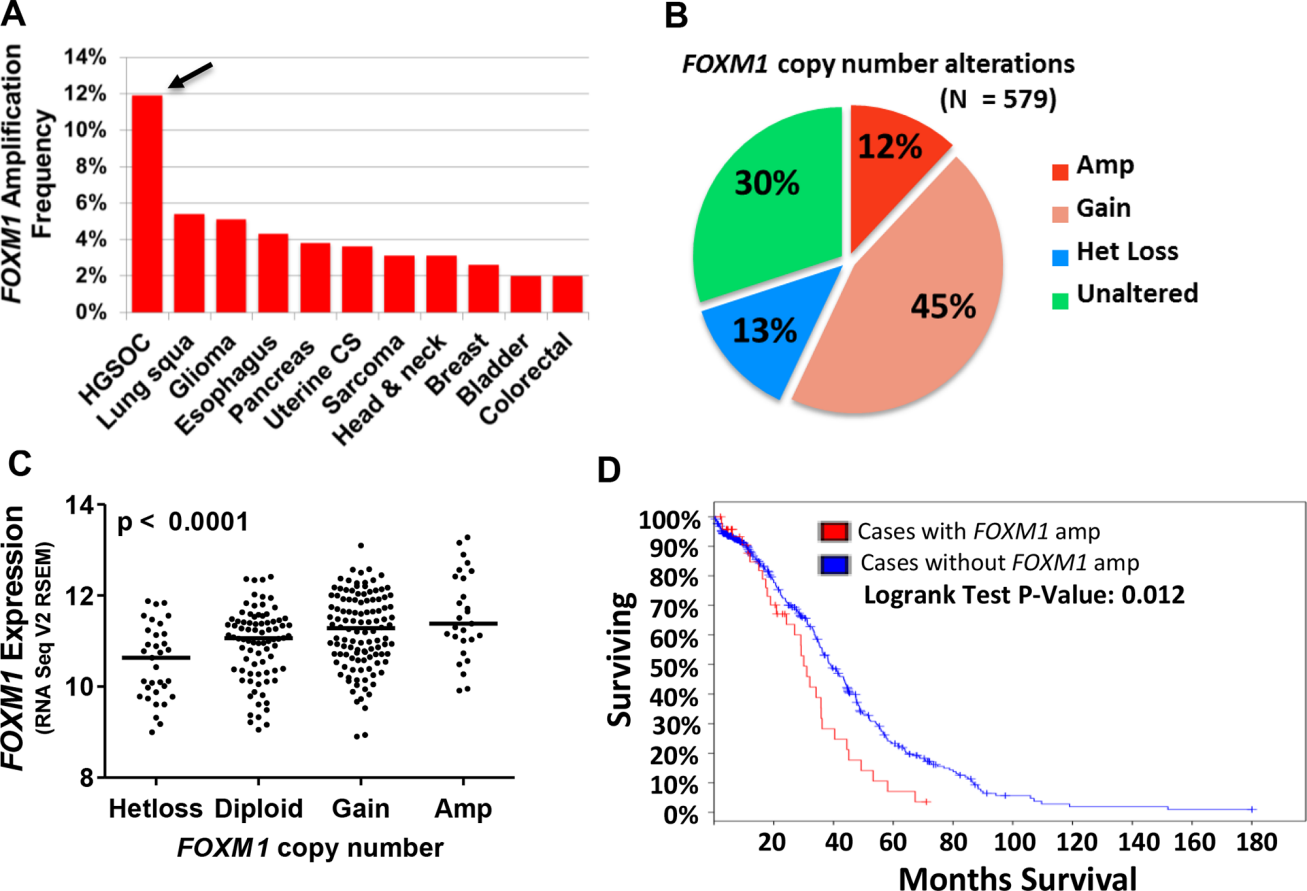
*FOXM1* copy number alterations (CNA) in HGSOC **A.** *FOXM1* amplification frequency in TCGA datasets. Arrow indicates HGSOC. **B.** *FOXM1* CNA in HGSOC TCGA datasets as determined by GISTIC. **C.** *FOXM1* expression (RNA Seq V2 RSEM, log2) compared to *FOXM1* copy number in HGSOC TCGA datasets. The *p* value for ANOVA with post-test for linear trend is shown. Lines represent group medians. **D.** Overall survival as a function of *FOXM1* amplification in HGSOC TCGA datasets. The *p* value for Logrank test is shown.

*FOXM1* is located at chromosome 12p13.33, a known amplified region in cancer [23, 33, 34]. We thus examined *FOXM1* copy number in TCGA datasets using cBioPortal [35, 36]. Notably, amongst all tumor types with TCGA data, *FOXM1* was most frequently amplified in HGSOC, with ~12% of tumors effected (Figure 1A). Together, over half of HGSOC cases showed either copy number gains or amplifications, suggesting FOXM1 as an HGSOC oncogene (Figure 1B). To determine if *FOXM1* copy number status correlates with expression, we compared *FOXM1* mRNA expression and copy number in TCGA HGSOC data. We observed a progressive increase in *FOXM1* expression with copy number status that was highly significant (Figure 1C). We additionally compared overall survival (OS) to *FOXM1* CNA and *FOXM1* expression, and observed that the former showed a significant correlation with OS, while the latter did not (Figure 1D and data not shown). This finding suggests that additional genes located at the amplified region of 12p13.33 may contribute to OS in HGSOC, and/or that FOXM1 protein or activation levels may be more relevant than mRNA levels for impacting OS. Finally, our analysis of TCGA mutational data did not reveal *FOXM1* mutations in HGSOC (data not shown).

### FOXM1 expression in relation to EOC type and progression status, and FOXM1 isoform expression in EOC

**Figure 2:**
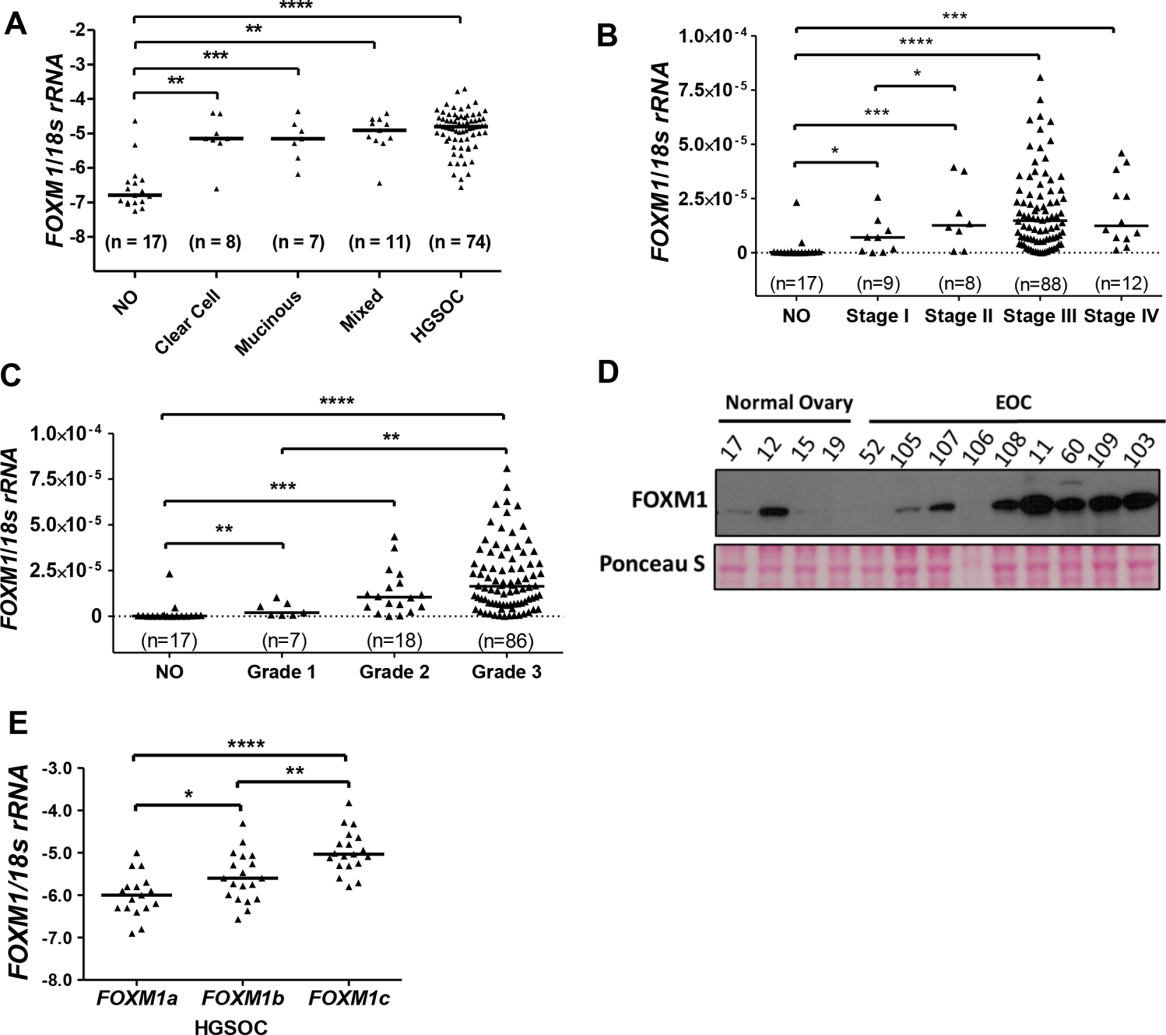
FOXM1 expression in EOC **A.** *FOXM1* expression measured with RT-qPCR (log10) in EOC histological subtypes as compared to normal ovary (NO). *FOXM1* expression was normalized to *18s rRNA*. **B.** *FOXM1* expression in NO and in EOC as a function of disease stage. **C.** *FOXM1* expression in NO and in EOC as a function of pathological grade. Lines represent group medians. Mann-Whitney test *p* values are shown. **D.** FOXM1 Western blot analysis in NO and EOC. Ponceau S staining is shown as a loading control. **E.** *FOXM1* isoform specific RT-qPCR (log10) measured in HGSOC tissues. Lines represent group medians. The Mann-Whitney test *p* value is shown. *p* value designation: **** < 0.0001, *** < 0.001, ** < 0.01, * < 0.05.

We next examined FOXM1 expression using an independent set of EOC tissues with diverse histology, stage, and grade [37, 38]. RT-qPCR analysis demonstrated that *FOXM1* is frequently overexpressed in different EOC histological subtypes relative to normal ovary (NO), and furthermore shows increased expression in both late-stage and high-grade disease (Figure 2A–2C). While we did not have mRNA from normal fallopian tube available for analysis, it is notable that the TCGA reported low expression of *FOXM1* mRNA in normal fallopian tube as compared to HGSOC (see FigS10.4 in [4]). Similar to the mRNA, FOXM1 protein expression was elevated in EOC as compared to NO (Figure 2D). FOXM1 has three known splice variants: *FOXM1a*, *b*, and *c*, which encode proteins with varying activities [24]. We used isoform specific RT-qPCR and found that *FOXM1c* is the predominant isoform expressed in EOC, followed by *FOXM1b* and *FOXM1a* (Figure 2E).

### *FOXM1* expression in HGSOC cell models

**Figure 3:**
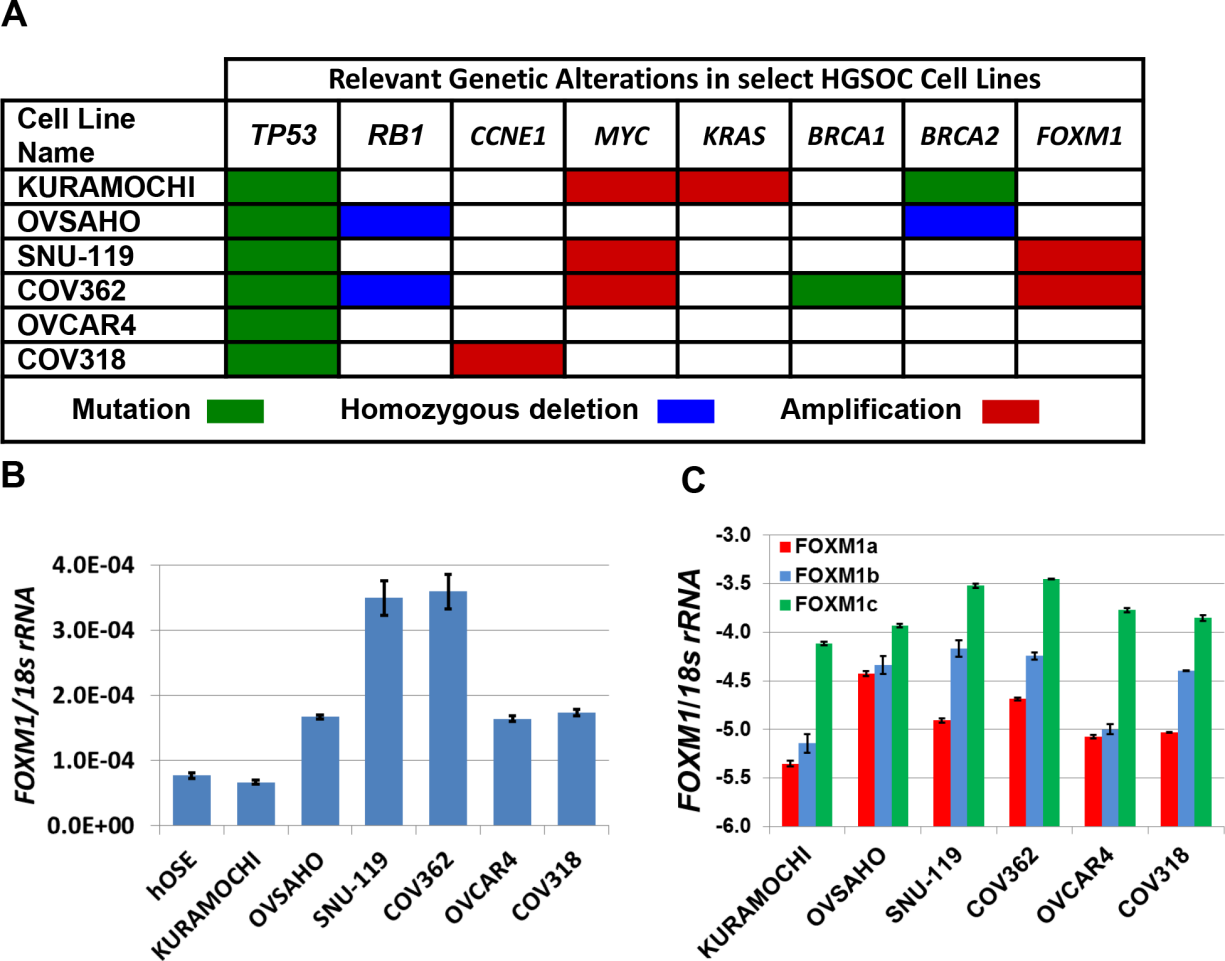
FOXM1 expression in HGSOC cell lines **A.** Relevant genetic alterations in HGSOC cell lines. Data were retrieved from CCLE and copy number alterations were visualized with IGV as described in *Methods*. **B.** Pan-*FOXM1* mRNA expression in HGSOC cell lines and hOSE cells (control) was measured by RT-qPCR. **C.** Isoform specific *FOXM1* mRNA expression in HGSOC cell lines was measured by RT-qPCR (log10). For B–C, bars represent mean ± SD.

We used clinically relevant cell models of human HGSOC to examine genetic influences on *FOXM1* expression [39]. All cell lines used have *TP53* mutations as well as additional genetic alterations relevant to HGSOC (Figure 3A). We found that *FOXM1* mRNA expression was elevated in all but one cancer cell line as compared to hOSE cells, and was heterogeneous in the HGSOC cell types (Figure 3B). Notably, highest *FOXM1* expression was observed in the two cell lines (SNU-119, COV362) in which the *FOXM1* locus is amplified. Isoform-specific RT-qPCR revealed highest expression of *FOXM1c*, moderate expression of *FOXM1b*, and lowest expression of *FOXM1a* in HGSOC cell lines. *FOXM1c* expression was highest in the SNU-119 and COV362 lines, in which *FOXM1* is amplified (Figure 3C). The relative expression of the three *FOXM1* isoforms is in agreement with our primary tumor data (Figure 2E).

### Disruption of Rb and p53 induces FOXM1 expression in murine and human OSE cells

**Figure 4:**
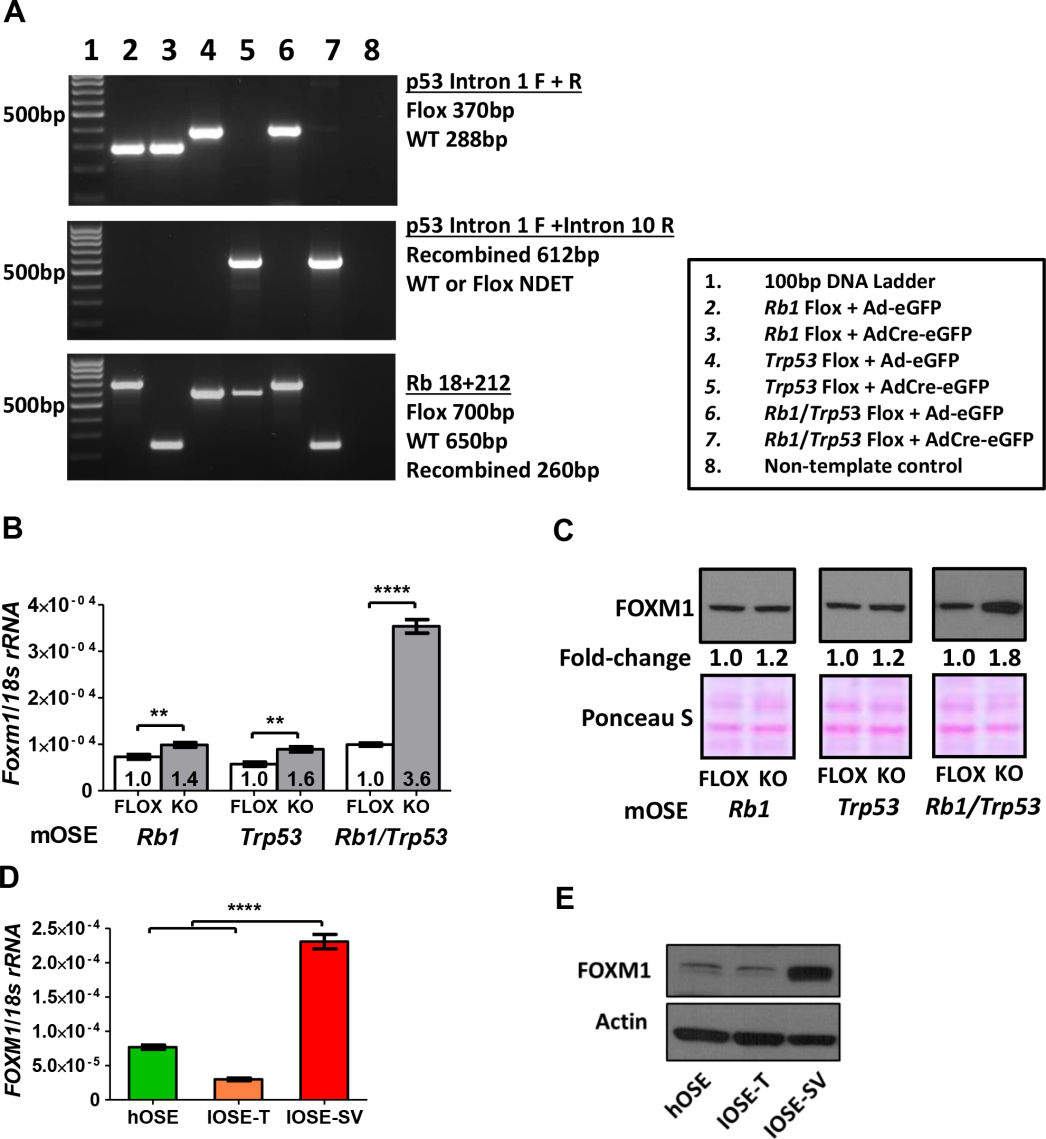
FOXM1 expression in murine and human OSE cells following Rb and/or p53 abrogation **A.** PCR genotyping of mOSE cells following infection with recombinant adenovirus expressing enhanced GFP (Ad-eGFP, control) or Cre recombinase + eGFP (AdCre-eGFP). **B–C.** FOXM1 expression in Rb and/or p53 floxed (control) and knockout (post-Cre infection) mOSE cells. B. *Foxm1* RT-qPCR with respective fold-change relative to the floxed control. Data represents mean ± SD. Students *t*-test *p* value is shown. C. FOXM1 Western blot with respective fold change relative to the floxed control, performed with nuclear lysates. Ponceau S staining is shown as a loading control. **D–E.** FOXM1 expression in primary and immortalized human OSE cells (hOSE, IOSE-T, IOSE-SV). Cell line descriptions are provided in the *Methods*. D. *FOXM1* RT-qPCR. Data represent mean ± SD. E. FOXM1 Western blot. β-actin is shown as a loading control. Students *t*-test *p* values: **** < 0.0001, *** < 0.001, ** < 0.01, * < 0.05.

The OSE is a potential tissue of origin for EOC, and primary OSE cells are useful for exploring EOC relevant processes [40, 41]. We first used established murine OSE (mOSE) cell models to examine mechanisms regulating FOXM1 expression. We focused on *TP53* and *RB1*, as mutations or disruptions in these genes are frequent in HGSOC [4, 9]. *Trp53* and *Rb1* knockout was achieved through Ad-Cre infection of mOSE cells as described previously (Figure 4A) [42]. While loss of either tumor suppressor gene (TSG) alone resulted in a modest upregulation of *Foxm1*, combined p53 and Rb loss led to robust induction (Figure 4B). Similar effects were observed for FOXM1 protein expression (Figure 4C). We next investigated the potential role of p53 and Rb in FOXM1 regulation in human OSE (hOSE) cells by measuring FOXM1 expression in hOSE cells immortalized with either SV40 Large T antigen (IOSE-SV), which leads to potent inactivation of p53 and Rb, or hTERT (IOSE-T), which leaves both proteins intact [43]. IOSE-SV cells showed significantly higher levels of expression of both FOXM1 mRNA and protein as compared to IOSE-T or primary (non-immortalized) human OSE cells (Figure 4D–4E). These data suggest that Rb and p53 play a major role in regulating FOXM1 expression in OSE cells.

### FOXM1 is overexpressed in murine ovarian cancer driven by combined p53/Rb1 disruption

**Figure 5:**
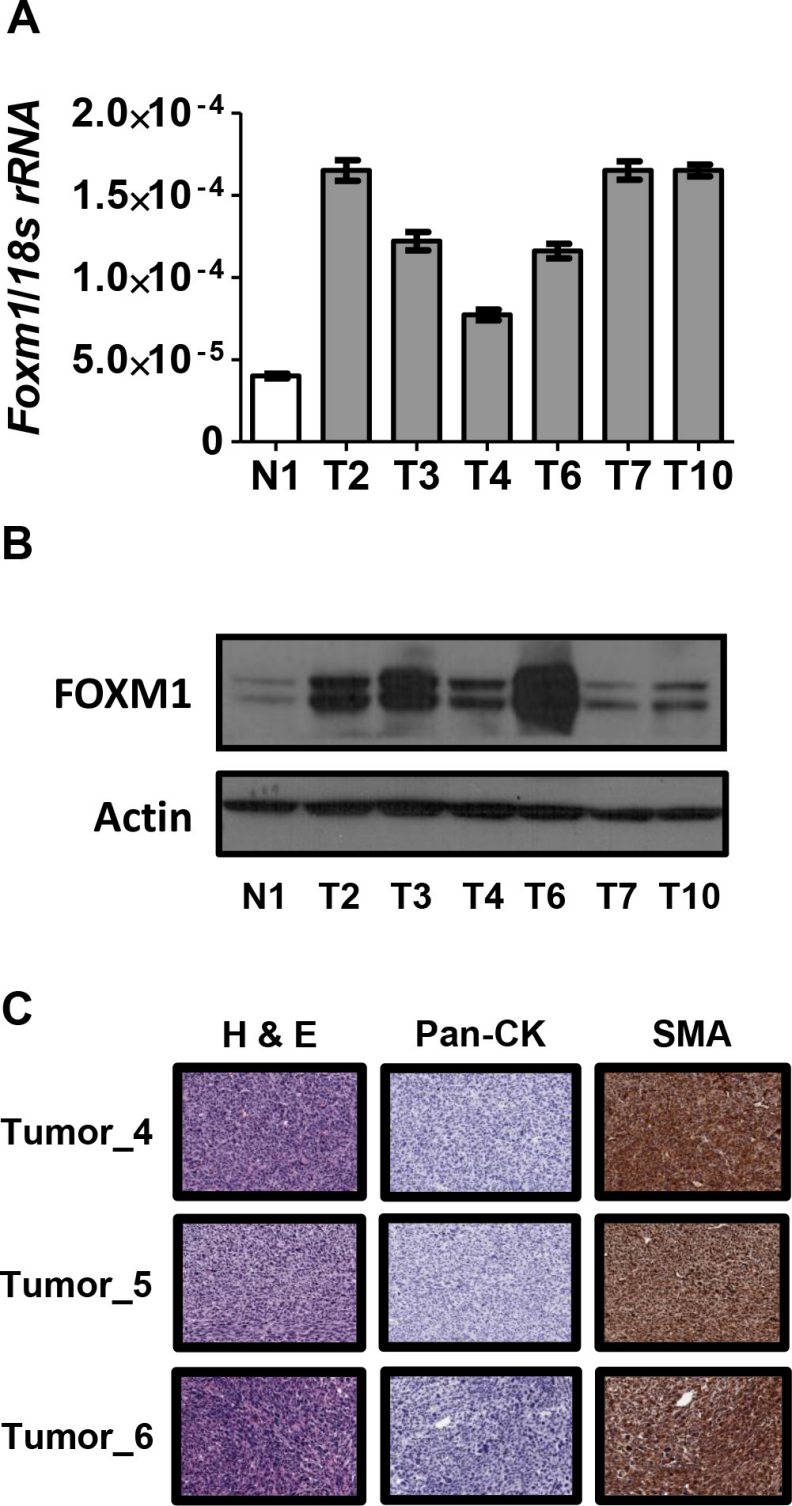
FOXM1 expression in Rb1/Trp53 knockout-driven murine ovarian cancer **A–B.** FOXM1 expression in *Rb1*/*Trp53* knockout murine ovarian tumor tissues (T) and murine normal ovary control tissue (N) The mouse model is described in *Methods*. A. *Foxm1* RT-qPCR. Data represents means ± SD. B. FOXM1 Western blot. β-actin is shown as a loading control. **C.** Ovarian tumor histology in Rb/p53 knockout mice. Paraffin sections of the tumors were stained with H&E or specific antibodies to pan-cytokeratin (Pan-CK) or smooth muscle actin (SMA). Images were captured using 20X magnification. Antigen detection is indicated by the presence of a brownish-red stain.

To complement the OSE cell studies, we measured FOXM1 expression in murine ovarian tumors developing after dual disruption of p53 and Rb in the OSE (see *Methods*). As shown in Figure 5A–5B, FOXM1 mRNA and protein expression were significantly increased in ovarian tumors as compared to the mouse normal ovary control. These *in vivo* data provide further support that loss of p53 and Rb contribute to FOXM1 overexpression in ovarian cancer. Notably, immunohistochemistry (IHC) analyses of the ovarian tumors arising in this model indicated that the tumors were negative for cytokeratin expression and positive for smooth muscle actin (Figure 5C). This finding suggests that cancer in this model may represent leiomyosarcoma, and not EOC, as reported previously [42].

### E2F1 and FOXM1 expression in OSE cells and EOC

**Figure 6:**
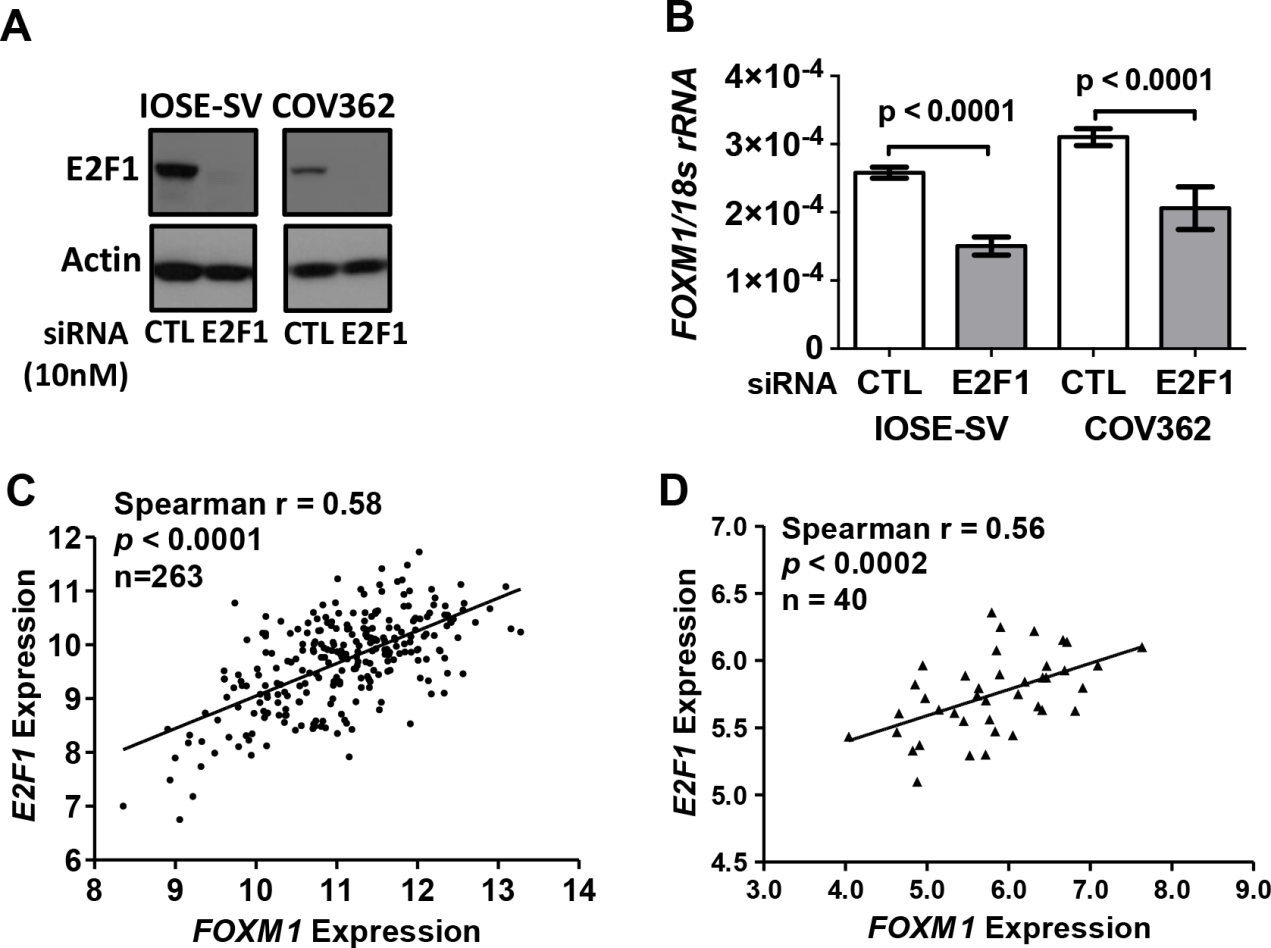
E2F1 and FOXM1 expression in IOSE-SV cells, HGSOC cells, and primary tumors **A–B.** siRNA knockdown of E2F1 (10 nM) in IOSE-SV and COV362 cells for 72 hours. A. E2F1 Western blot. β-actin is shown as a loading control. B. *FOXM1* RT-qPCR, normalized to *18s rRNA*. Data represent mean ± SD. Student's *t*-test *p* value is shown. **C–D.** *E2F1* and *FOXM1* expression correlation in human EOC. C. Correlation in 263 HGSOC tissues from TCGA datasets (gene expression determined by RNA seq V2, log2). D. Correlation in an independent set of 40 EOC tissues (gene expression determined by Affymetrix HG 1.0ST microarray, log2).

Transcriptional activation of *FOXM1* following Rb loss suggests that E2F transcription factors may contribute to FOXM1 overexpression. To test this, we used IOSE-SV and COV362 cells, which have high FOXM1 expression as well as alterations in p53 and Rb. Following E2F1 knockdown by siRNA (Figure 6A), *FOXM1* mRNA expression in both cell types was significantly reduced, as compared to the non-targeting siRNA control (Figure 6B). To validate this finding in the primary disease setting, we tested whether *FOXM1* correlates with *E2F1* expression in human EOC. As shown in Figure 6C–6D, in both the TCGA HGSOC dataset and in our independent set of EOC tissues, expression of *FOXM1* and E2F1 were highly correlated. Together, these data implicate E2F1 in promoting FOXM1 expression in EOC.

### Functional contribution of FOXM1 to EOC cell cycle progression and target gene expression

**Figure 7:**
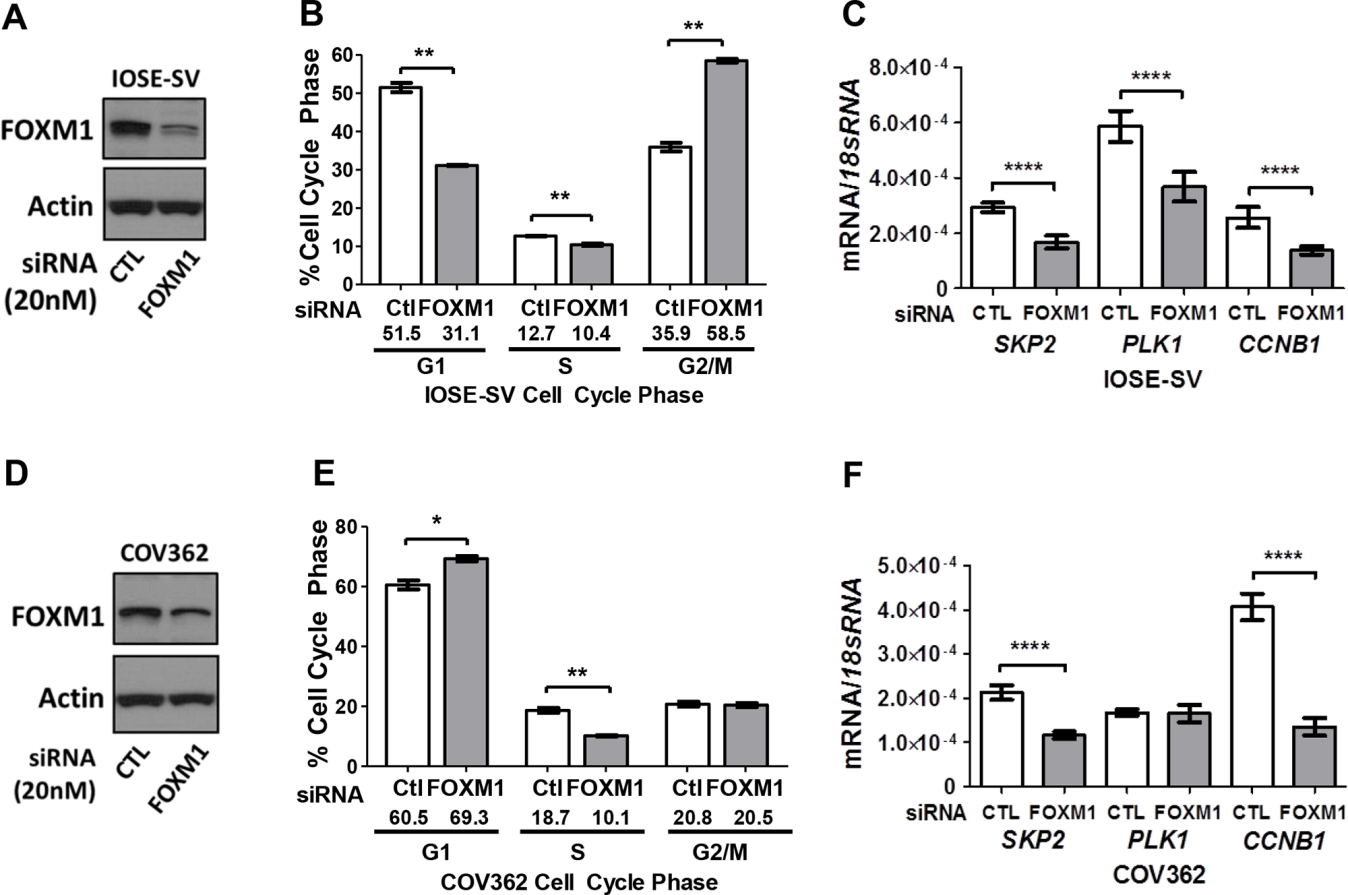
Impact of FOXM1 knockdown on cell cycle progression and target gene expression in IOSE-SV and COV362 cells Transient siRNA-mediated knockdown of FOXM1 (20 nM) was completed for 72 hours. **A.** Validation of FOXM1 protein knockdown in IOSE-SV cells. FOXM1 protein expression was determined by Western blot, and β-actin is shown as a loading control. **B.** Cell cycle analysis of IOSE-SV cells following FOXM1 or control siRNA treatment. **C.** FOXM1 target gene expression determined by RT-qPCR in IOSE-SV cells, following FOXM1 or control siRNA treatment. Expression data are shown for *SKP2*, *PLK1*, and *CCNB1*, each normalized to *18s rRNA*. **D–F.** Same as A-C, except the experiment was performed using COV362 cells. Bars represent mean ± SD. Student's *t* test *p* values are shown. *P* value designation: **** < 0.0001, *** < 0.001, ** < 0.01, * < 0.05.

To determine if FOXM1 plays a functional role in EOC cells, we explored its canonical function in cell cycle progression using knockdown and overexpression approaches. Knockdown of FOXM1 was efficient in IOSE-SV cells (Figure 7A), and led to accumulation of cells in G2-M, with concomitant decreases in both G1 and S (Figure 7B; representative histograms shown in Supplementary Figure 1A). In COV362 cells, FOXM1 knockdown also led to decreased cells in S phase, but caused accumulation of cells in G1 with no significant alteration of G2-M (Figure 7D–7E; representative histograms shown in Supplementary Figure 1B). To determine whether the observed effect of FOXM1 knockdown on cell cycle progression coincided with altered expression of relevant FOXM1 target genes, we analyzed *SKP2*, *PLK1*, and *CCNB1* expression. SKP2 promotes G1-S transition, while PLK1 and CCNB1 promote G2-M transition, both downstream of FOXM1 [28, 31, 44]. In agreement with our cell cycle data, FOXM1 knockdown in IOSE-SV and COV362 downregulated these genes, with the lone exception of *PLK1* in COV362 (Figure 7C, 7F).

**Figure 8:**
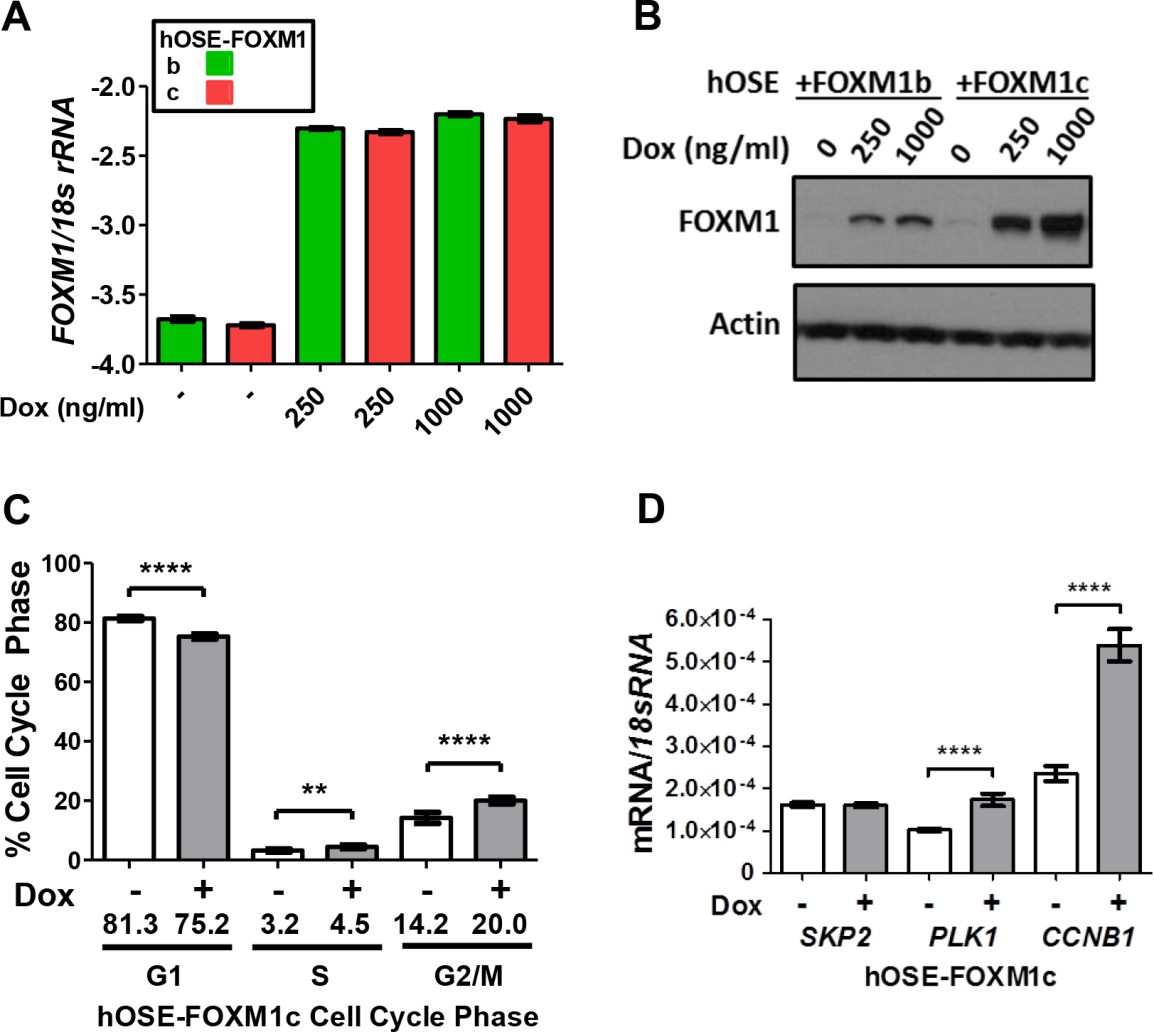
Impact of FOXM1 overexpression on cell cycle progression and target gene expression in hOSE cells **A–B.** Dox-inducible FOXM1b and FOXM1c overexpression in primary hOSE cells after 72 hours of doxycycline treatment as indicated. A. *FOXM1* RT-qPCR (log10). B. FOXM1 Western blot. β-actin is shown as a loading control. **C.** Cell cycle analysis following Dox-inducible FOXM1c overexpression in primary hOSE cells after 72 hours of treatment. Cells treated with 250 ng/ml and 1000 ng/ml doxycycline were combined for analysis and compared against the control without treatment. **D.** FOXM1 target gene expression was measured by RT-qPCR in hOSE cells following 72 hours of doxycycline treatment to induce FOXM1c. Expression data are shown for *SKP2*, *PLK1*, and *CCNB1*, each normalized to *18s rRNA*. Data represents mean ± SD. Student's *t*-test *p* values are shown. *P* value designation: **** < 0.0001, *** < 0.001, ** < 0.01, * < 0.05.

In addition to FOXM1 knockdown, we overexpressed FOXM1b or FOXM1c using a stable doxycycline (Dox)-inducible system in primary hOSE cells. Interestingly, while the mRNA expressions were identical, the FOXM1c protein appeared to be more stable than FOXM1b in these cells (Figure 8A–8B). FOXM1c overexpression in hOSE led to increased cells in S and G2/M, with a decrease in G1 (Figure 8C). In contrast, overexpression of FOXM1b did not alter cell cycle (data not shown). To determine whether the effect of FOXM1c overexpression on cell cycle coincided with altered expression of relevant FOXM1 target genes, we again analyzed *SKP2*, *PLK1*, and *CCNB1*. Overexpression of FOXM1c in hOSE cells led to upregulation of *PLK1* and *CCNB1*, while *SKP2* was unaffected (Figure 8D). These data are consistent with the functional impact of FOXM1 in EOC cell cycle regulation, and suggest that this activity may be mediated by FOXM1's function as a transcriptional regulator. Although the effect of FOXM1c overexpression on cell cycle was modest, this could be due to the primary hOSE cell model used. In agreement, the effect of FOXM1 overexpression on cell cycle progression in primary hOSE are reminiscent of that reported in cancer cells, although the effects were more robust in the latter [45, 46].

## DISCUSSION

Several mechanisms have been reported to contribute to FOXM1 overexpression in cancer, including gene amplification, loss of negative regulation by p53, Rb, and FOXO3, and transcriptional activation by E2F and Myc [13–17, 22, 23]. To date, the mechanisms underlying FOXM1 upregulation in HGSOC have not been described, although FOXM1 pathway activation is extremely frequent in this malignancy. Here we demonstrate that, in EOC, *FOXM1* is upregulated at the transcriptional level by combined loss of Rb and p53, and show that FOXM1 copy number gains correlate with increased FOXM1 expression in primary tumors and cell lines. Combinatorial loss of p53 and Rb in murine and human OSE cells synergistically induced FOXM1 expression, and murine ovarian cancer arising in a p53/Rb compound deletion model led to FOXM1 overexpression. In addition, we demonstrate that E2F1 contributes to FOXM1 overexpression in cell models, and closely correlates with FOXM1 expression in primary tumors. Thus, our data establish p53 and Rb as negative regulators, and E2F1 and copy number gain as positive regulators, of FOXM1 expression in EOC. Consistent with our p53 data, it was recently shown that Nutlin 3-mediated p53 activation repressed FOXM1 in EOC cells [47].

We observed that p53 and Rb loss cooperatively drive high level FOXM1 expression in EOC relevant cell models, and our data reveal E2F1 as a factor contributing to this induction. Several potential mechanisms may underline these observations. First, loss of Rb function leads to activation of E2F transcription factors [48], and two putative E2F sites have been identified in the FOXM1 promoter [22]. Second, the Rb-E2F pathway is regulated by p21, a potent negative regulator of cyclin-dependent kinases (CDK) and a direct transcriptional target of p53. Therefore, functional loss of p53 may relieve p21-mediated repression of E2F1, which in turn may promote FOXM1 expression. In agreement, prior work shows that p53-mediated repression of FOXM1 is partially p21-dependent [14, 22]. p53-mediated negative regulation of FOXM1 may also be independent of effects on the Rb-E2F pathway, although this remains to be determined. In addition to p53, Rb, and E2F1, other relevant mechanisms of FOXM1 induction involve Myc and FOXO3 [16, 17]. These may act independently or in concert with p53 and Rb loss, and require further study using EOC models.

Importantly, a recent study observed increased FOXM1 staining in early precursor Serous Tubal Intraepithelial Carcinoma (STIC) lesions, and showed that FOXM1 expression was maintained in invasive tumors [16]. As *TP53* mutations appear to be a ubiquitous early event in human HGSOC, we speculate that during HGSOC tumor progression, loss of *Rb* function and/or *FOXM1* amplification, coupled with the p53 impairment already present, leads to high level FOXM1 expression. Consistent with this model, our data indicate that FOXM1 expression is markedly elevated in late stage, high-grade EOC. Further verification of this model requires determination of FOXM1 protein expression during EOC disease progression.

We found that the predominant *FOXM1* isoform expressed in HGSOC is *FOXM1c*. An earlier study showed that FOXM1c is the predominant isoform expressed in pancreatic cancer, while another study showed that FOXM1b is the major isoform expressed in other cancer types [20, 49]. FOXM1c has alternative exon A1; residues in this region can be phosphorylated by the RAF/MEK/MAPK signaling cascade, providing a distinction with FOXM1b [50]. Considering the differential expression and functional potential of different FOXM1 isoforms, it is important to determine which variants are responsible for the oncogenic activity in EOC. In this context, our cell cycle data suggests that FOXM1c, but not FOXM1b, drives cell cycle progression in hOSE cells. Notably, a recent study discovered the expression of additional isoforms of FOXM1 in ovarian cancer, and speculated that these isoforms may be constitutively active [51].

In the current study, we demonstrated a role for FOXM1 in cell cycle progression using primary and immortalized human OSE cells and using a HGSOC cell line. Beyond its role in cell cycle progression, FOXM1 has been shown to contribute to other important oncogenic phenotypes in ovarian cancer, including platinum and taxane resistance, epithelial-to-mesenchymal transition (EMT), cell migration, and cell invasion [52–55]. Additionally, it is plausible that FOXM1 overexpression, combined with p53 gain of function mutations, may synergistically promote genomic instability in EOC. For example, FOXM1 upregulation induced genomic instability in normal human keratinocytes, and FOXM1 is a member of a conserved gene expression profile for genomic instability in human cancer [56, 57]. Furthermore, p53 gain of function mutations can positively regulate FOXM1 and correlate with higher levels of genomic instability as compared to p53 null mutations [47, 58]. Based on the functions of FOXM1 that have been described, it is likely that FOXM1 contributes to multiple oncogenic phenotypes during HGSOC genesis and progression, including genomic instability in early STIC lesions, EMT in primary tumors, and metastatic tumor growth and drug resistance in late stage disease.

Based on the oncogenic role of FOXM1 in cancer, there is significant interest in developing drugs that target this protein. This is particularly relevant in HGSOC, for which current therapeutic regimens, especially for late-stage disease, are inadequate. Until recently, available FOXM1 inhibitors, i.e. the thiazole antibiotics Siomycin A and Thiostrepton, were non-specific and had global effects on proteasome-dependent pathways [59, 60]. However, a recent paper identified and characterized a specific inhibitor of FOXM1, FDI-6 [61]. FDI-6 was reported to specifically inhibit the DNA binding activity of FOXM1, but not other FOX family members, and was shown to inhibit cancer cell growth *in vitro*. FDI-6 needs additional validation, as concerns have been raised about its specificity [62], and its potency may require improvement for possible treatment of FOXM1-dependent cancers. Despite these caveats, the existence of a small molecule inhibitor of FOXM1 provides a new and exciting opportunity to pursue relevant translational studies in FOXM1-dependent cancers, including HGSOC.

## MATERIALS AND METHODS

### The cancer genome atlas (TCGA) data analysis

TCGA provisional data was retrieved from cBioPortal on January 5, 2015. All provisional cancer datasets were analyzed for FOXM1 mutation and somatic copy-number alterations. The genomic profile of FOXM1 was further analyzed in the HGSOC (Ovarian Serous Cystadenocarcinoma-TCGA Provisional) dataset for putative somatic copy-number alterations from GISTIC [63], using Onco Query Language (OQL), and mRNA expression (RNA seq V2 RSEM). GISTIC predicts gene copy number alterations according to sample specific thresholds generated by comparing chromosomal segments with median chromosomal arm copy numbers. High gains (Amp) are segments with copy number that exceed the maximum median chromosomal arm copy number for that sample by at least 0.1; low gains (Gain) are segments with copy numbers from 2.1 to the high gain threshold; neutral segments (Diploid) have copy numbers between 1.9 and 2.1; shallow losses (Hetloss) have copy numbers between 1.9 and the deep deletion threshold; and deep deletions (Homdel) have copy numbers that are below the minimum median chromosomal arm copy number for that sample by at least 0.1. Overall patient survival was determined by Kaplan-Meier Survival. E2F1 mRNA expression (RNA seq V2 RSEM) was retrieved from the same dataset. All parameters were set at default.

### Human primary tissues

Normal ovary (NO) and epithelial ovarian cancer (EOC) tissues were obtained from patients undergoing surgical resection at Roswell Park Cancer Institute (RPCI) under Institutional Review Board-approved protocols, and were described previously [37, 38]. Frozen tissues were processed for biochemical extractions as described previously [37, 38].

### Reverse transcriptase quantitative PCR (RT-qPCR)

Total RNA was purified using TRIzol (Invitrogen) and quality was determined by RNA denaturing gel. Briefly, one μg of RNA was DNase-treated using the DNA-free kit (Ambion), and converted to cDNA using the iScript cDNA synthesis kit (BioRad). One μl of 1:5 cDNA sample dilutions were used for qPCR reactions. Standard curves were prepared using gel-purified end-point RT-PCR products. All samples were run in triplicate, and all gene expression data were normalized to *18s rRNA*. PCR was performed with an annealing temperature of 60°C and a total of 45 cycles for all primer pairs. Dissociation curves were performed to confirm specific product amplification. RT-qPCR standards for each gene were generated from a mixture of human or mouse cell cDNA via end point RT–PCR. Gradient PCR reactions were used to optimize annealing temperatures for each primer set. Primer sequences are listed in Supplementary Table S1.

### Western blot analyses

Whole cell protein extracts were prepared with RIPA buffer [1X PBS, 1% NP40, 0.5% sodium deoxycholate, 0.1% sodium dodecyl sulfate (SDS)] supplemented with protease and phosphatase inhibitors (Sigma), and centrifuged at 4°C for 10 minutes at 14000*g*. Nuclear extracts were prepared using the NE-PER Nuclear and Cytoplasmic Extraction Kit (Thermo Scientific) supplemented with protease and phosphatase inhibitors. Protein concentration was determined by the BCA protein assay (Thermo Scientific). Equal amounts of protein (30–50 μg) were fractionated on 4–12% gradient SDS-polyacrylamide gel electrophoresis gels (Invitrogen) and transferred to PVDF membrane (Roche). Membranes were stained with Ponceau S to confirm efficient transfer and equal loading then blocked with 5% nonfat dry milk in Tris-buffered saline Tween-20 (TBST) for 1 hour at room temperature. Membranes were incubated with primary antibodies in 5% nonfat dry milk in TBST at 4°C overnight followed by incubation with secondary antibody in 5% nonfat dry milk in TBST for 1 hour at room temperature. The following primary antibodies, purchased from Santa Cruz Biotechnology, were used at the indicated dilutions: FOXM1 (sc-500; 1:500, sc-271746; 1:500), E2F1 (sc-251; 1:500), β-Actin (sc-47778; 1:5000). Enhanced chemiluminescence (Thermo Fisher Scientific) was used for protein detection. Quantification of protein expression was performed using ImageJ software (Image Processing and Analysis in Java, National Institute of Health) [64].

### The Cancer Cell Line Encyclopedia (CCLE)

The copy-number and mutational profiles of CCLE cell lines KURAMOCHI, SNU-119, OVSAHO, COV362, COV318 and OVCAR4 were visualized using the Integrative Genomics Viewer (IGV, version 1.4.2.) [65].

### Cell culture

COV362 and COV318 cell lines (Sigma) were cultured in DMEM (Corning) supplemented with 10% fetal bovine serum (FBS, Invitrogen), 2 mM glutamine (Life Technologies), 1% penicillin-streptomycin (pen-step, Life Technologies). KURAMOCHI and OVSAHO (Japanese Collection of Research Bioresources Cell Bank) and SNU-119 (Korean Cell Line Bank) cell lines were cultured in RPMI-1640 (Hyclone) supplemented with 10% FBS and 1% pen-strep. OVCAR4 cells (National Cancer Institute Division of Cancer Treatment and Diagnosis Cell Line Repository) were cultured in RPMI-1640 supplemented with 10% FBS and 1% pen-strep. Primary hOSE cells (ScienCell) were cultured in Ovarian Epithelial Cell Medium (ScienCell, 7311). IOSE-T (a.k.a. IOSE-21, hOSE immortalized with hTERT) cells [43] were a generous gift from Professor Francis Balkwill (Cancer Research UK) and were cultured in Medium 199/MCDB105 (1:1, Sigma) supplemented with 15% FBS, 1% pen-strep, 10 ng/mL human epidermal growth factor (Life Technologies), 0.5 μg/mL hydrocortisone (Sigma), 5 μg/mL bovine insulin (Cell Applications), 34 μg protein/mL bovine pituitary extract (Life Technologies). IOSE-SV (a.k.a. IOSE-121, hOSE immortalized with SV40 Large T antigen) cells were a generous gift from Dr. Nelly Auersperg (University of British Columbia) and were cultured in Medium 199/MCDB105 (1:1) supplemented with 10% FBS and 25 μg/ml gentamicin (Life Technologies). mOSE cells [42] were a generous gift from Professor Barbara Vanderhyden (Ottawa Hospital Research Institute) and were cultured in Alpha Modified MEM (Corning) containing 10% FBS, 0.05% pen-strep, 1 μg/ml gentamicin, and 1% insulin–transferrin–sodium–selenite solution (ITSS, Roche). HEK293T cells (American Type Tissue Culture Collection) were cultured in DMEM with 10% FBS and 1% pen-strep. All cell lines were maintained at 37°C in a humidified incubator with 5% CO2. Cell culture medium was changed every 3–5 days depending on cell density. For routine passage, cells were split at a ratio of 1:3–10 when they reached 85% to 90% confluence.

### Adenoviral transduction of mOSE cells and p53/Rb genotyping

Recombinant adenovirus expressing enhanced GFP (Ad-eGFP, control), or both eGFP and Cre recombinase (AdCre-eGFP), were purchased from the University of Iowa Gene Transfer Vector Core. mOSE cells were transduced at an MOI of 200 for 6 hours. Media was changed and cells were allowed to expand for 10 days before harvesting for analysis. Genomic DNA was isolated using the Puregene Tissue Kit (Qiagen). DNA was re-suspended in Tris-EDTA (50 mM, pH 6.8). Genotyping for *p53* and *Rb* genes were performed as previously described [42]. PCR was performed with an annealing temperature of 60°C and 30 cycles for all primer pairs. Primer sequences are listed in Supplementary Table S1.

### Murine ovarian cancer transgenic model

*Trp5*3^loxP/loxP^/*Rb1*^loxP/loxP^ mice (floxed *Trp53* and *Rb1*) were a kind gift from Professor Kenneth Gross (RPCI). All mice were maintained identically, following recommendations of the Institutional Laboratory Animal Use and Care Committee (RPCI). Intrabursal injections of recombinant adenovirus expressing both enhanced GFP and Cre recombinase (AdCre-eGFP) or eGFP alone (Ad-eGFP) as the contralateral control (University of Iowa Gene Transfer Vector Core) were performed on adult mice in estrus as previously described [66]. Mice were determined to be in estrus by vaginal cytology. The original viral stock solution was diluted with PBS to 3.5 × 10^9^ pfu/mL immediately before injection of 10 μL. Mice were euthanized and subjected to necropsy when tumor mass exceeded 1 cm or the animal exhibited other signs of sickness, such as abdominal distension and moribund behavior. Tumor samples were dissected and snap frozen in liquid nitrogen and stored at −80°C. Frozen tumor samples were ground into a powder with a mortar and pestle over liquid nitrogen and immediately processed for RNA (miRNeasy Mini Kit, Qiagen) and whole cell protein cell extracts were prepared with RIPA buffer (1X PBS, 1% NP40, 0.5% sodium deoxycholate, 0.1% SDS) supplemented with protease and phosphatase inhibitors, and centrifuged at 4°C for 10 minutes at 14000*g*. RNA was treated for contaminating DNA using the TURBO DNA-free Kit (Ambion), and integrity was determined using a bioanalyzer (Agilent). One ug of DNase-treated RNA was converted to cDNA using the iSCRIPT cDNA Synthesis Kit (Bio-Rad).

### Immunohistochemistry

Formalin-fixed paraffin blocks were cut into 4 μm sections, placed on charged slides, and dried at 60°C for one hour. Slides were cooled to room temperature, deparaffinized in three changes of xylene, and rehydrated using graded alcohols. For antigen retrieval, slides were heated in a steamer for 20 min in citrate buffer pH = 6 (BioCare Medical, B910) for Smooth Muscle Actin or target retrieval solution pH = 9 (Dako, S2367) for Cytokeratin and allowed to cool for 20 min, endogenous peroxidase quenched with aqueous 3% H2O2 for 10 minutes and washed with PBS/T. Slides were loaded on a Dako autostainer and serum free protein block (Dako, X0909) was applied for 5 minutes, blown off and the corresponding antibody was applied. Smooth Muscle Actin antibody (Abcam, ab5694) was applied at 1:125 (Rabbit IgG) for one hour. Powervision poly HRP anti-rabbit IgG (Leica; catalog #PV6119) was then applied for 30 minutes. L- DAB (Leica; catalog #PV6126) applied for 5 minutes, was used for chromogen visualization. Pan-Cytokeratin antibody (Dako, Z0622) was applied at 1:1750 (Rabbit IgG) for one hour. Rabbit Envision/ labeled polymer HRP anti-rabbit (Dako; catalog #K4003) was then applied for 30 minutes. DAB (Dako; catalog #K3468) applied for 10 minutes, was used for chromogen visualization. Lastly, the slides were counterstained with Hematoxylin, rinsed, and cover slipped.

### E2F1 and FOXM1 siRNA knockdown

siRNAs (Supplementary Table S1) were transfected with Lipofectamine RNAiMax reagent (Life Technologies), according to the manufacturer's instructions, using the concentrations indicated in the Figures. The non-targeting siRNA #2 (Dharmacon) was used as a control as described previously [67]. 72 hours after transfection, cells were prepared for total RNA and protein extractions, and cell cycle analyses.

### Microarray analysis of FOXM1 and E2F1 expression

Affymetrix HG 1.0ST arrays were used to determine the expression of *FOXM1* and *E2F1* in EOC. Probe generation, array hybridization, and expression analyses were performed by the Next Generation Sequencing and Expression Analysis Core Facility at the University at Buffalo Center for Excellence in Bioinformatics. Samples included 40 EOC.

### Cell cycle analyses

Cell cycle analysis was performed utilizing the Muse Cell Analyzer (EMD Millipore) and Cell Cycle Assay Kit (EMD Millipore), following the manufacturer's instruction. Briefly, sub-confluent cells were trypsinized, washed with PBS, and filtered with 37 μm mesh cap tube then fixed in ice-cold 70% ethanol while vortexing and stored 18–24 hours at −20°C. Cells were then stained for 30 minutes at room temperature with propidium iodide (PI) containing RNase, and immediately processed for cell cycle analysis. Representative DNA content histograms are shown in Supplementary Figure S1.

### Inducible FOXM1 expression in hOSE cells

The tetracycline-inducible lentiviral pCW57.1-HA-FOXM1b and pCW57.1-DDK-FOXM1c vectors were generated by subcloning human *FOXM1b* and *FOXM1c* from pCMV6 (Origene: SC112825 and SC128214) into pCMV6-AN-HA or –AN-DDK plasmids (Origene: PS100013 and PS100014), respectively, then subcloning into the pCW57.1 (Addgene: 41393) with Gateway cloning (Life Technologies). All plasmids were sequence verified. Replication-deficient lentivirus expressing Dox-inducible FOXM1 was produced by transient transfection of 6.0 μg psPAX2 (Addgene: 12260), 2.0 μg pMD2.G (Addgene: 12259), and 8.0 μg transfer plasmid into HEK293T cells in a 10-cm dish with Lipofectamine 2000 reagent (Life Technologies), according to the manufacturer's instructions. Viral supernatants were collected at 48 hours, passed through a 0.45-μm filter, and titered by serial dilution with puromycin (Life Technologies) selection and colony formation. The highest dilution producing drug selected colonies was used to transduce primary hOSE cells in the presence of polybrene (4 μg/ml, Sigma), and 0.5 μg/ml puromycin was introduced 48 hours post-infection. After five days of puromycin selection, cells were allowed to recover and expand for one week. Cells were seeded in 6-well plates and the next day media was changed with or without doxycycline (Sigma) to induce transgene expression. Media with or without doxycycline was changed every 24 hours. After 72 hours, cells were prepared for total RNA and protein extractions, and cell cycle analyses.

### Statistical analyses

Student's *t*-test was used to compare differences between means between two groups. Mann-Whitney test was used to compare differences between medians between two groups. One-way analysis of variance (ANOVA) with a post-test for linear tend was used to compare two or more groups. For all analyses, significance was inferred at *p* < 0.05 and *p* values were two-sided. Graphpad Prism statistical software (GraphPad Software, Inc).

## SUPPLEMENTARY TABLE AND FIGURE



## References

[1] Cannistra S.A (2004). Cancer of the ovary. The New England journal of medicine.

[2] Vaughan S (2011). Rethinking ovarian cancer: recommendations for improving outcomes. Nature reviews Cancer.

[3] Bast R.C, Hennessy B, Mills G.B (2009). The biology of ovarian cancer: new opportunities for translation. Nat Rev Cancer.

[4] Cancer Genome Atlas Research, N (2011). Integrated genomic analyses of ovarian carcinoma. Nature.

[5] Kuo K.T (2009). Analysis of DNA copy number alterations in ovarian serous tumors identifies new molecular genetic changes in low-grade and high-grade carcinomas. Cancer Res.

[6] Ciriello G (2013). Emerging landscape of oncogenic signatures across human cancers. Nature genetics.

[7] Etemadmoghadam D (2013). Synthetic lethality between CCNE1 amplification and loss of BRCA1. Proc Natl Acad Sci U S A.

[8] Karst A.M (2014). Cyclin E1 deregulation occurs early in secretory cell transformation to promote formation of fallopian tube-derived high-grade serous ovarian cancers. Cancer Res.

[9] Patch A.M (2015). Whole-genome characterization of chemoresistant ovarian cancer. Nature.

[10] Myatt S.S, Lam E.W (2007). The emerging roles of forkhead box (Fox) proteins in cancer. Nat Rev Cancer.

[11] Chen X (2013). The forkhead transcription factor FOXM1 controls cell cycle-dependent gene expression through an atypical chromatin binding mechanism. Mol Cell Biol.

[12] Halasi M, Gartel A.L (2013). FOX(M1) news--it is cancer. Mol Cancer Ther.

[13] Pandit B, Halasi M, Gartel A.L (2009). p53 negatively regulates expression of FoxM1. Cell cycle.

[14] Barsotti A.M, Prives C (2009). Pro-proliferative FoxM1 is a target of p53-mediated repression. Oncogene.

[15] McGovern U.B (2009). Gefitinib (Iressa) represses FOXM1 expression via FOXO3a in breast cancer. Mol Cancer Ther.

[16] Levanon K, Sapoznik S (2014). FOXO3a loss is a frequent early event in high-grade pelvic serous carcinogenesis. Oncogene.

[17] Blanco-Bose W.E (2008). C-Myc and its target FoxM1 are critical downstream effectors of constitutive androstane receptor (CAR) mediated direct liver hyperplasia. Hepatology.

[18] Mencalha A.L (2012). Forkhead box M1 (FoxM1) gene is a new STAT3 transcriptional factor target and is essential for proliferation, survival and DNA repair of K562 cell line. PLoS One.

[19] Teh M.T (2002). FOXM1 is a downstream target of Gli1 in basal cell carcinomas. Cancer Res.

[20] Kong X (2013). Dysregulated expression of FOXM1 isoforms drives progression of pancreatic cancer. Cancer Res.

[21] Xia L.M (2009). Transcriptional up-regulation of FoxM1 in response to hypoxia is mediated by HIF-1. J Cell Biochem.

[22] Millour J (2011). ATM and p53 regulate FOXM1 expression via E2F in breast cancer epirubicin treatment and resistance. Mol Cancer Ther.

[23] Yu J (2011). Array-based comparative genomic hybridization identifies CDK4 and FOXM1 alterations as independent predictors of survival in malignant peripheral nerve sheath tumor. Clin Cancer Research.

[24] Korver W (1997). The winged-helix transcription factor Trident is expressed in cycling cells. Nucleic Acids Res.

[25] Korver W (1997). The winged-helix transcription factor Trident is expressed in actively dividing lymphocytes. Immunobiology.

[26] Park HJ (2008). Anaphase-promoting complex/cyclosome-CDH1-mediated proteolysis of the forkhead box M1 transcription factor is critical for regulated entry into S phase. Mole Cell Biology.

[27] Anders L (2011). A systematic screen for CDK4/6 substrates links FOXM1 phosphorylation to senescence suppression in cancer cells. Cancer Cell.

[28] Wang I.C (2005). Forkhead box M1 regulates the transcriptional network of genes essential for mitotic progression and genes encoding the SCF (Skp2-Cks1) ubiquitin ligase. Mole Cell Biol.

[29] Wang I.C (2008). FoxM1 regulates transcription of JNK1 to promote the G1/S transition and tumor cell invasiveness. J Biol Chem.

[30] Fu Z (2008). Plk1-dependent phosphorylation of FoxM1 regulates a transcriptional programme required for mitotic progression. Nat Cell Biol.

[31] Laoukili J (2005). FoxM1 is required for execution of the mitotic programme and chromosome stability. Nature Cell Biol.

[32] Wonsey D.R (2005). Loss of the forkhead transcription factor FoxM1 causes centrosome amplification and mitotic catastrophe. Cancer Res.

[33] Delahaye-Sourdeix M (2015). The 12p13.33/RAD52 Locus and Genetic Susceptibility to Squamous Cell Cancers of Upper Aerodigestive Tract. PLoS one.

[34] Green M.R (2011). Integrative genomic profiling reveals conserved genetic mechanisms for tumorigenesis in common entities of non-Hodgkin's lymphoma. Genes Chromosomes Cancer.

[35] Gao J (2013). Integrative analysis of complex cancer genomics and clinical profiles using the cBioPortal. Sci Signal.

[36] Cerami E (2012). The cBio cancer genomics portal: an open platform for exploring multidimensional cancer genomics data. Cancer Discov.

[37] Woloszynska-Read A (2007). DNA methylation-dependent regulation of BORIS/CTCFL expression in ovarian cancer. Cancer Immun.

[38] Woloszynska-Read A (2011). Coordinated cancer germline antigen promoter and global DNA hypomethylation in ovarian cancer: association with the BORIS/CTCF expression ratio and advanced stage. Clin Cancer Res.

[39] Domcke S (2013). Evaluating cell lines as tumour models by comparison of genomic profiles. Nat Commun.

[40] McCloskey C.W (2014). A new spontaneously transformed syngeneic model of high-grade serous ovarian cancer with a tumor-initiating cell population. Front Oncol.

[41] Garson K (2012). Technical challenges and limitations of current mouse models of ovarian cancer. J Ovarian Res.

[42] Clark-Knowles K.V (2009). Conditional inactivation of Brca1, and Rb in mouse ovaries results in the development of leiomyosarcomas. PLoS one.

[43] Li N.F (2007). Human ovarian surface epithelial cells immortalized with hTERT maintain functional pRb and p53 expression. Cell Prolif.

[44] Leung T.W (2001). Over-expression of FoxM1 stimulates cyclin B1 expression. FEBS Letters.

[45] Wang Z (2007). Down-regulation of Forkhead Box M1 transcription factor leads to the inhibition of invasion and angiogenesis of pancreatic cancer cells. Cancer Res.

[46] Chan D.W (2008). Over-expression of FOXM1 transcription factor is associated with cervical cancer progression and pathogenesis. J Pathol.

[47] Zhang X (2014). Targeting of mutant p53-induced FoxM1 with thiostrepton induces cytotoxicity and enhances carboplatin sensitivity in cancer cells. Oncotarget.

[48] Harbour J.W, Dean D.C (2000). The Rb/E2F pathway: expanding roles and emerging paradigms. Genes Dev.

[49] Lam A.K (2013). FOXM1b, which is present at elevated levels in cancer cells, has a greater transforming potential than FOXM1c. Front Oncol.

[50] Ma R.Y (2005). Raf/MEK/MAPK signaling stimulates the nuclear translocation and transactivating activity of FOXM1c. J Cell Sci.

[51] Barrett C.L (2015). Systematic transcriptome analysis reveals tumor-specific isoforms for ovarian cancer diagnosis and therapy. Proc Natl Acad Sci U S A.

[52] Chiu WT (2015). FOXM1 confers to epithelial-mesenchymal transition, stemness and chemoresistance in epithelial ovarian carcinoma cells. Oncotarget.

[53] Zhao F (2014). Overexpression of Forkhead Box Protein M1 (FOXM1) in Ovarian Cancer Correlates with Poor Patient Survival and Contributes to Paclitaxel Resistance. PLoS one.

[54] Zhou J (2014). FOXM1 modulates cisplatin sensitivity by regulating EXO1 in ovarian cancer. PLoS one.

[55] Wen N (2014). Overexpression of FOXM1 predicts poor prognosis and promotes cancer cell proliferation, migration and invasion in epithelial ovarian cancer. J Transl Med.

[56] Teh MT (2010). Upregulation of FOXM1 induces genomic instability in human epidermal keratinocytes. Mol Cancer.

[57] Carter SL (2006). A signature of chromosomal instability inferred from gene expression profiles predicts clinical outcome in multiple human cancers. Nat Genet.

[58] Hanel W, Moll U.M Moll (2012). Links between mutant p53 and genomic instability. J Cell Biochem.

[59] Wang M (2011). Micelle-encapsulated thiostrepton as an effective nanomedicine for inhibiting tumor growth and for suppressing FOXM1 in human xenografts. Mole Cancer Ther.

[60] Radhakrishnan S.K (2006). Identification of a chemical inhibitor of the oncogenic transcription factor forkhead box M1. Cancer Res.

[61] Gormally M.V (2014). Suppression of the FOXM1 transcriptional programme via novel small molecule inhibition. Nat Commun.

[62] Kalinichenko V.V, Kalin T.V (2015). Is there potential to target FOXM1 for ‘undruggable’ lung cancers?. Expert Opin Ther Targets.

[63] Beroukhim R (2007). Assessing the significance of chromosomal aberrations in cancer: methodology and application to glioma. Proc Natl Acad Sci U S A.

[64] Schneider C.A, Rasband W.S, Eliceiri K.W (2012). NIH Image to ImageJ: 25 years of image analysis. Nat Methods.

[65] Robinson J.T (2011). Integrative genomics viewer. Nat Biotechnol.

[66] Flesken-Nikitin A (2003). Induction of carcinogenesis by concurrent inactivation of p53 and Rb1 in the mouse ovarian surface epithelium. Cancer Res.

[67] Link P.A (2009). Distinct roles for histone methyltransferases G9a and GLP in cancer germ-line antigen gene regulation in human cancer cells and murine embryonic stem cells. Mole Cancer Res.

