# Supplementary material for: Genetic determinants of FOXM1 overexpression in epithelial ovarian cancer and functional contribution to cell cycle progression
Source: Oncotarget. 2015 Jul 16;6(29):27613–27. doi: 10.18632/oncotarget.4546 (PMC4695012; doi:10.18632/oncotarget.4546)
Supplement: Supplementary file 1 [file oncotarget-06-27613-s001.pdf]

# SUPPLEMENTARY TABLE AND FIGURE

## Supplementary Table S1: Barger *et al*

| Mouse RT-qPCR | | |
|---|---|---|
| **Primer Name** | **Primer Sequence** | **Reference** |
| FOXM1 F | 5′ AGCGTTAAGCAGGAACTGGA 3′ | [1] |
| FOXM1 R | 5′ GGAAGTGGTCCTCAATCCAA 3′ | |
| 18s rRNA F | 5′ ATGGCCGTTCTTAGTTGGTG 3′ | [2] |
| 18s rRNA R | 5′ GAACGCCACTTGTCCCTCTA 3′ | |
| **Mouse PCR Genotyping** | | |
| **Primer Name** | **Primer Sequence** | **Reference** |
| p53_int1_new_F | 5′ CACA**A**AAAAACAGGTTAAA**C**CCAG 3′ | Modified from [3] as indicated. |
| p53_int1 R | 5′ AGCACATAGGAGGCAGAGAC 3′ | [3] |
| p53_int10 F | 5′ AAGGGGTATGAGGGACAAGG 3′ | |
| p53_int10 R | 5′ GAAGACAGAAAAGGGGAGGG 3′ | |
| Rb212 F2 | 5′ GAAAGGAAAGTCAGGGACATTGGG 3′ | |
| Rb18 R | 5′ GGCGTGTGCCATCAATG 3′ | |
| **Human RT-qPCR** | | |
| **Primer Name** | **Primer Sequence** | **Reference** |
| FOXM1a F | 5′ TGGGGAACAGGTGGTGTTTGG 3′ | [4] |
| FOXM1a R | 5′ GCTAGCAGCACTGATAAACAAAG 3′ | |
| FOXM1c F | 5′ CAATTGCCCGAGCACTTGGAATCA 3′ | |
| FOXM1c R | 5′ TCCTCAGCTAGCAGCACCTTG 3′ | |
| FOXM1b F | 5′ CCAGGTGTTTAAGCAGCAGA 3′ | |
| FOXM1b R | 5′ TCCTCAGCTAGCAGCACCTTG 3′ | |
| FOXM1 F | 5′ GCAGGCTGCACTATCAACAA 3′ | [5] |
| FOXM1 R | 5′ TCGAAGGCTCCTCAACCTTA 3′ | |
| SKP2 F | 5′ GGTGTTTGTAAGAGGTGGTATCGC 3′ | [6] |
| SKP2 R | 5′ CACGAAAAGGGCTGAAATGTTC 3′ | |
| CCNB1 F | 5′ AACTTTCGCCTGAGCCTATTTT 3′ | [7] |
| CCBN1 R | 5′ TTGGTCTGACTGCTTGCTCTT 3′ | |
| PLK1 F | 5′-GGACTATTCGGACAAGTACG-3′ | [8] |
| PLK1 R | 5′-CGGAAATATTTAAGGAGGGTGA-3′ | [9] |
| 18s rRNA F | 5′ CAGCCACCCGAGATTGAGCA-3′ | |
| 18s rRNA R | 5′ TAGTAGCGACGGGCGGTGTG 3′ | |
| **Human siRNA Knockdown** | | |
| **siRNA Name** | **siRNA Sequence** | **Reference** |
| E2F1 | 5′ GUCACGCUAUGAGACCUCA 3′ | [10] |
| FOXM1 | 5′ GGACCACUUUCCCUACUUU 3′ | [6] |

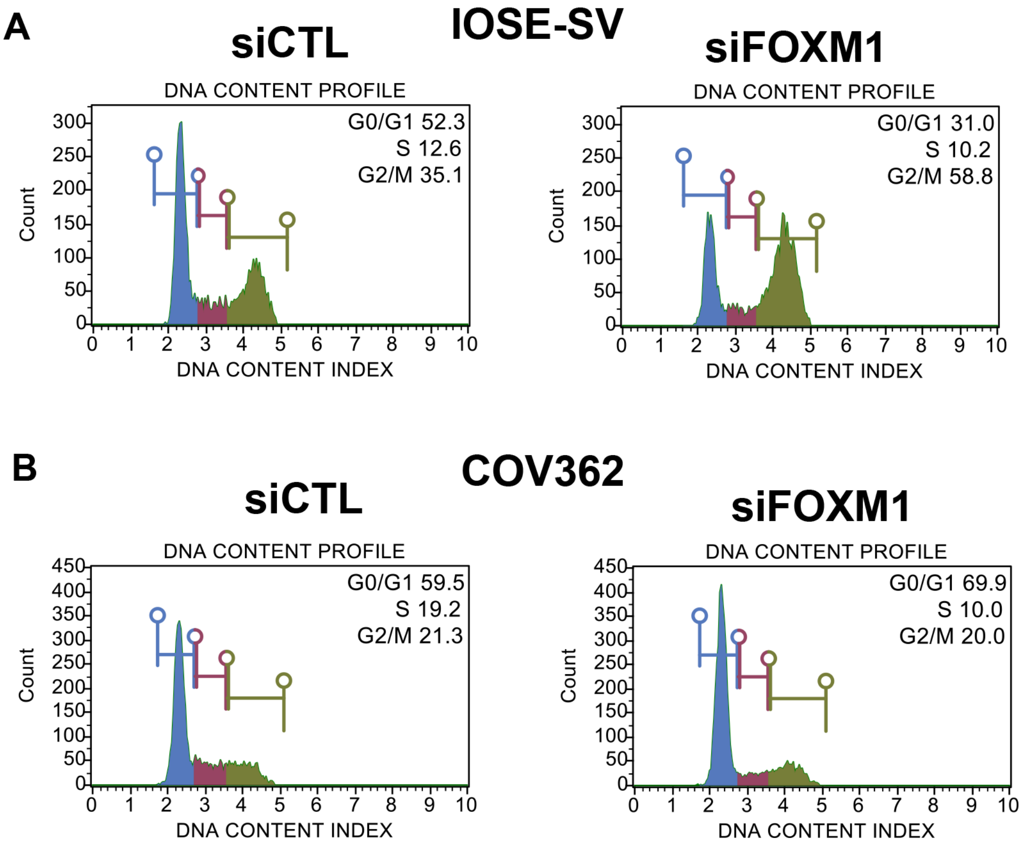


**Supplementary Figure S1: Representative cell cycle histograms following FOXM1 siRNA knockdown.** Cells were treated with a non-targeting siRNA (siCTL) or siRNA targeting FOXM1 (siFOXM1) (20nM) for 72 hours. **A.** IOSE-SV cells. **B.** COV362 cells.

## REFERENCES


1. Wang Z, et al. FoxM1 in tumorigenicity of the neuroblastoma cells and renewal of the neural progenitors. Cancer Res. 2011; 71:4292–302.
2. Wang N, et al. The LIM-only factor LMO4 regulates expression of the BMP7 gene through an HDAC2-dependent mechanism, and controls cell proliferation and apoptosis of mammary epithelial cells. Oncogene. 2007; 26:6431–41.
3. Clark-Knowles K.V, et al. Conditional inactivation of Brca1, and Rb in mouse ovaries results in the development of leiomyosarcomas. PLoS One. 2009; 4:e853.
4. Gemenetzidis E, et al. FOXM1 upregulation is an early event in human squamous cell carcinoma and it is enhanced by nicotine during malignant transformation. PLoS One. 2009; 4:e4849.
5. Carr J.R, et al. FoxM regulates mammary luminal cell fate. Cell Rep. 2012; 1:715–29.
6. Wang I.C, et al. Forkhead box M1 regulates the transcriptional network of genes essential for mitotic progression and genes encoding the SCF (Skp2-Cks1) ubiquitin ligase. Mol Cell Biol. 2005; 25:10875–94.
7. Wang H, et al. EPS8 upregulates FOXM1 expression, enhancing cell growth and motility. Carcinogenesis. 2010; 31:1132–41.
8. Amato A, et al. RNAi mediated acute depletion of retinoblastoma protein (pRb) promotes aneuploidy in human primary cells via micronuclei formation. BMC Cell Biol. 2009; 10:79.
9. Todd P.K, et al. Histone deacetylases suppress CGG repeat-induced neurodegeneration via transcriptional silencing in models of fragile X tremor ataxia syndrome. PLoS Genet. 2010; 6:e1001240.
10. Rogoff H.A, et al. Apoptosis associated with deregulated E2F activity is dependent on E2F1 and Atm/Nbs1/Chk2. Mol Cell Biol. 2004; 24:2968–77.
